# Building confianza, sharing Consejos: A grounded theory study of Latine mothers' socialization of coping

**DOI:** 10.1111/jora.70319

**Published:** 2026-11-09

**Authors:** Virnaliz Jimenez, Kelly M. Tu

**Affiliations:** ^1^ Department of Human Development and Family Studies University of Illinois Urbana‐Champaign Urbana Illinois USA

**Keywords:** coping, cultural values, familism, grounded theory, Latino families, parenting, respect

## Abstract

Adolescence is a critical developmental period characterized by common stressors such as academic pressures and changing family and peer dynamics that may be exacerbated by additional stressors, such as acculturative stress, language barriers, and discrimination, for Latine youth. Cultural values of familismo (familism; loyalty to family) and respeto (respect; obedience, deference to authority) may play an important role in Latine parents' socialization of coping (SOC) messages about how to manage stressful situations, with implications for Latine youths' coping and well‐being. Yet, culturally specific approaches to parents' SOC in the literature are scarce. Toward theory building via grounded theory methods, this study focused on how Latine parents socialize youth to cope with interpersonal and academic stressors with a sample of 23 majority Mexican‐origin participants: 12 mothers (*M*_age_ = 40.08 years) and 11 adolescents (*M*_age_ = 14.36 years; 18% boys, 82% girls). Mothers and adolescents each participated in a one‐time, 1.5‐h semi‐structured interview (Spanish or English). Mothers shared their experiences in providing advice regarding coping with interpersonal and academic stress. The resulting analysis presents a theoretical model of how Latine mothers guide youth to cope with stress and the role of cultural values (familismo, respeto) in this process. Results highlight the central role of fomentando confianza (building trust) as a foundational relational context enabling Latine mothers to guide their adolescents in managing stressors and mothers' advice of prioritizing relational harmony, refocusing expectations, encouraging effort, and redefining agency, all of which are embedded within cultural values. This grounded theory model refines existing theories of parenting and coping socialization and advances understanding of relational contexts and cultural processes that may support Latine adolescent well‐being.

In the United States (U.S.), Latine youth (a gender neutral term to describe individuals of Latin American origin) not only face typical adolescent stressors such as changing family dynamics and peer relationships as well as academic pressures (Bravo et al., 2021), but also unique stressors such as acculturative stress, language barriers, discrimination (Brietzke & Perreira, 2017), and a hostile sociopolitical climate stemming from anti‐immigrant policies (Suárez‐Orozco et al., 2021). Collectively, these challenges may place Latine youth at higher risk for mental health problems (Torres et al., 2022). Toward identifying processes through which to promote Latine youth well‐being, the current study aimed to investigate Latine mothers' cultural values and their socialization of coping—or efforts to help their youth cope with stressors.

Parent socialization of coping (SOC) messages provide youth with specific suggestions on how to cope with stressful situations (e.g., problem‐solving; Kliewer et al., 1994), and evidence indicates that parents' coping suggestions are associated with youth coping (Abaied & Rudolph, 2011; Anderson et al., 2019) and youth mental health outcomes (Abaied & Rudolph, 2010). Yet, much of this work has been conducted with predominantly white samples, identifying a gap in our understanding of ethnic minority parents' SOC processes. Such work is needed because the normative interpersonal and academic challenges experienced by ethnic minority youth are compounded by sociocultural factors.

An important consideration in this work is the cultural values that may shape parenting practices used to guide adolescents on how to respond to stress. Indeed, two cultural values have been linked with Latine youth mental health and well‐being, including familismo (familism), emphasizing strong family bonds, and respeto (respect), which underscores obedience and deference to authority (Halgunseth, 2019). Direct connections between parents' SOC and cultural values remain a gap in the literature, despite evidence demonstrating how cultural values shape youth well‐being through parenting (Calzada et al., 2010; Hernández & Bámaca‐Colbert, 2016).

Toward addressing this gap, the current study utilized grounded theory methodology to explain how Latine mothers socialize youth to cope with interpersonal and academic stress, which are particularly salient among Latine youth (Cross et al., 2019; Irwin et al., 2022). Such findings may have implications for providing insights about how to better support Latine mothers as they help their adolescents navigate challenges.

## STRESSORS AND DEVELOPMENTAL CONTEXTS OF LATINE YOUTH

Adolescence involves increasing autonomy, changing family and peer relationships, and growing academic demands (Bravo et al., 2021). For Latine adolescents, these normative developmental tasks may intersect with culturally and structurally situated stressors related to family relationships, immigration, language, discrimination, and educational inequities (Brietzke & Perreira, 2017). These contexts may shape the stressors youth encounter and the support and coping guidance they receive from parents. For instance, at the interpersonal level, Latine adolescents may experience family stressors associated with family obligations, acculturation gaps (e.g., parent–child differences in strength of orientation towards heritage culture and U.S. mainstream culture), and intergenerational conflict (Brietzke & Perreira, 2017; Garcia & Lindgren, 2009). Latine adolescents may also encounter peer stress, including conflict and bullying, with approximately 18% of Latino youth reported having been bullied at school (e.g., rumors, insults, being shoved; Irwin et al., 2022), which may stem, in part, from discrimination (Brietzke & Perreira, 2017).

Regarding education, Latine adolescents' academic stressors, such as not understanding assignments and/or struggling to complete schoolwork, which is common for many youth at some point during their educational journey, may be further exacerbated by educational and immigration policies, language barriers, and family expectations. For instance, English‐only policies may be especially challenging for immigrant youth whose first language is not English (Behnke et al., 2010), or Deferred Action for Childhood Arrivals policies that can affect Latine adolescents' educational experiences and opportunities (Espinoza‐Herold & González‐Carriedo, 2017; Suárez‐Orozco et al., 2021). Moreover, Latine youth grapple with high educational expectations from their parents (Camacho‐Thompson et al., 2019; Cross et al., 2019) and are encouraged to view education as a key pathway to future opportunities and upward mobility (Cross et al., 2019; Ovink, 2013), as well as a way to honor family obligations and contribute to collective well‐being (Ovink, 2013). Taken together, Latine adolescents may experience heightened academic challenges and pressure.

In the context of these stressors, youth coping—intentional efforts to regulate their emotions, cognitions, behaviors, or environment—can have implications for their well‐being (e.g., Compas et al., 2001, 2017). Thus, it is important to not only understand the types of stressors that Latine youth may uniquely experience and how they cope with such stressors, but also how parents may support the development of youth coping via their socialization of coping efforts. Even during adolescence, parents continue to play a key role as sources of guidance as youth learn to respond to stress.

## PARENT SOCIALIZATION OF COPING: INTEGRATING CULTURAL VALUES

Parents can play a role in shaping youths' responses to stress through multiple family processes (Kliewer et al., 1994). Drawing from the socialization of coping framework, this study focused on the parental coaching path, which refers to parents' socialization of coping messages that provide youth with suggestions on how to cope with stressful situations via specific actions and/or appraisals of threat (Kliewer et al., 1994). Parents' SOC suggestions have been conceptualized into two broad categories: (1) engagement coping *suggestions* towards addressing the stressor directly (i.e., problem‐solving, support‐seeking) or adapting to the stress context (i.e., positive thinking) and (2) disengagement coping *suggestions* that promote avoidance, distraction, denial, or wishful thinking (e.g., Abaied & Rudolph, 2010, 2011). Parents' SOC efforts have been examined in relation to youths' experiences of general stressors, as well as specific stressors, including peer (Abaied & Rudolph, 2010, 2011; Tu et al., 2021) and academic (Tu et al., 2024) stressors.

Investigations of parent SOC that have included Latine families have been limited, particularly with regard to interpersonal stressors. Two studies with predominantly White families, but that included ~3%–6% Hispanic/Latino youth and/or parents, examined the link between parent SOC (in relation to peer stress) and youth mental health and found that parents' engagement and disengagement coping suggestions were associated with lower internalizing problems, but only under certain conditions—such as under conditions of low peer stress or for girls only (Anderson et al., 2021; Watson et al., 2022; for other studies with predominantly Black or White samples, see Abaied & Rudolph, 2010, 2011; Anderson et al., 2019; Jimenez & Tu, 2025; Tu et al., 2021). With academic stressors, Latine families similarly have limited representation. In two studies drawing from the same sample of families (~10%–15% Hispanic/Latino), parents' engagement (e.g., problem‐solving, help‐seeking, cognitive reappraisal) and disengagement coping suggestions for dealing with academic challenges were identified via self‐reports (Tu et al., 2020) and observations from mother–youth conversations (Tu et al., 2024). Additionally, a few correlations emerged, linking mothers' and fathers' engagement coping suggestions with youths' more adaptive coping with academic challenges (Tu et al., 2020). Although some Latine families were included in the aforementioned studies, the pattern of findings may not reflect the cultural nuances and parenting dynamics that characterize Latine families.

Indeed, according to Calzada et al.'s (2010) cultural framework of Latino parenting, cultural influences shape parenting processes and, in turn, child functioning. Attention to culture in SOC processes may therefore be especially important for understanding parenting among Latine families. As Latine parents navigate U.S. mainstream culture, they do so in relation to their heritage culture, the values and practices rooted in their families' countries of origin and community histories (Calzada et al., 2010). Within this framework, acculturative status, or the extent to which families may maintain their heritage culture, may influence the values they seek to cultivate in their children, and cultural socialization provides one pathway through which these values are conveyed. Cultural socialization involves parents' efforts to transmit cultural beliefs, expectations, traditions, and knowledge to their children (Halgunseth, 2019). For example, parents who place more emphasis on maintaining heritage culture (i.e., enculturation) may be more likely to endorse and socialize core Latino values such as familismo compared to parents who place more emphasis on U.S. mainstream values (i.e., acculturation) such as independence. These differing socialization goals may then be reflected in distinct parenting practices, including how they communicate expectations, guide children's behavior, and respond to children's emotions and stress.

Extending work that has tested Calzada et al.'s (2010) model in examining links between cultural values and parenting *styles* (e.g., authoritative, authoritarian parenting; Chen et al., 2020; Santisteban et al., 2012), we focused specifically on the role of cultural values in relation to parenting *practices* or the behaviors used to socialize youth (Smetana, 2011). Some emerging research has linked endorsement of familismo and respeto with parenting practices, including parental involvement and supervision. For example, Latine and Mexican‐origin parents who endorse stronger familismo and respeto values tend to demonstrate more involved parenting (Santisteban et al., 2012) and more parental monitoring (for sons but not daughters; Chen et al., 2020), respectively.

Yet, our understanding of how Latine cultural values, such as familismo and respeto, are linked to parent SOC processes is limited. Considering Calzada et al.'s (2010) cultural framework with Kliewer et al.'s (1996) SOC framework might suggest that Latine parents' coping suggestions may be more nuanced than a distinction between engagement and disengagement coping suggestions. Parents' messages may simultaneously address a stressful experience and communicate family expectations. In this way, culture may shape the broader SOC process as well as the content of the advice. Thus, we propose that considering a family's adherence to their heritage culture is essential, as cultural values can inform how parents understand stress, what they view as effective coping, and how they guide youth in ways that support positive development and well‐being.

Prior work has shown that attending to culture can reveal patterns and processes not captured by existing frameworks (Domenech Rodríguez et al., 2009). Moreover, much of the existing literature on Latine parenting has emphasized broad parenting styles (e.g., authoritarian, authoritarian) and Calzada et al.'s (2010, 2013) framework has predominantly been tested with younger children, leaving open questions about how these cultural influences operate for adolescents. Focusing on parenting practices in the context of adolescents' everyday stressors is important because these practices are proposed to have a direct effect on youth outcomes (Darling & Steinberg, 1993). Thus, our study responds to calls for an expanded understanding of parenting practices within Latine families (Halgunseth, 2019).

In summary, despite the growing parent SOC literature, attention to SOC processes among culturally diverse families has been limited. Such work is needed to clarify how SOC operates across diverse cultural backgrounds and to better identify pathways that promote youth well‐being, particularly in relation to common, yet salient, daily interpersonal and academic stressors that are compounded by sociocultural factors (e.g., discrimination; Torres et al., 2022). Thus, work that explores parents' SOC in ways that reflect families' priorities and cultural values, such as emphasizing the centrality of family, can deepen and enrich the SOC literature.

## THE PRESENT STUDY

In this study, Latine refers to individuals of Latin American origin or descent, while pan‐ethnic labels can obscure distinctions in race, class, national origin, and citizenship, we use this gender‐neutral alternative to Latina/o because it adheres more closely to Spanish grammar and reflects our commitment to inclusive language. The existing literature on parent SOC among Latine families is scarce, revealing gaps regarding parent socialization of coping among more culturally diverse families and how parents' goals and cultural context may shape their coping messages to youth (Darling & Steinberg, 1993; Halgunseth, 2019). In attending to cultural values that may illuminate culturally grounded parenting strategies that are overlooked by pre‐existing models or frameworks, this study sought to identify Latine cultural influences on the parent socialization of coping process using Grounded Theory methods (GTM; Charmaz, 2014). GTM is used by researchers interested in generating theory about social processes across different contexts (Charmaz, 2014). Our work initially drew upon the culturally informed model of Latino parenting (Calzada et al., 2010, 2013) and the socialization of coping framework (Kliewer et al., 1994) with the goal of using GTM to develop a model through which to address our overarching research question: How do Latine parents socialize youth to cope with interpersonal and academic stress?

## METHODS

A constructivist grounded theory approach (Charmaz, 2014) guided this study to theorize how Latine parents guide youth in coping with interpersonal and academic stressors. This approach assumes that knowledge is contextually grounded and co‐constructed between researchers and participants (Charmaz, 2014; Creswell & Creswell, 2017). Accordingly, we recognized our positionalities as integral to the knowledge produced and sought to situate parental socialization of coping within its broader social and cultural context. We actively engaged with participants' perspectives, striving to capture a wide range of meanings and to illuminate the circumstances in which these practices unfolded.

### Authors' positionality

Guided by the Framework of Researcher Racial and Cultural Positionality (Milner, 2007), this study engaged in ongoing reflexivity surrounding the first author's positionality as a cisgender Black, Latine woman, born and raised in the U.S. by immigrant parents from the Dominican Republic. Her experiences growing up in a rural, predominantly immigrant community, shaped her sensitivity to discrimination, resource scarcity, and the importance of culture, race, and immigration in family life. These experiences and prior work with Latine families informed her assumptions, for instance, that immigrant parents may frame coping in relation to navigating structural barriers and discrimination. Acknowledging her simultaneous positioning as an insider and outsider across different Latine subgroups, the first author engaged in continuous reflection through reflexive journaling, memo writing, and collaborative discussions with research assistants to mitigate assumptions of shared experiences and privilege participants' own narratives. Two Mexican American undergraduate research assistants collaborated with the first author in the initial phase of the study and model refinement, offering insights that enriched the analytic process.

The second author is a cisgender Asian American woman, born in the U.S. to Chinese parents who immigrated from Vietnam. Her research examines parenting and adolescent stress regulation in peer and academic contexts. Motivated by the first author's commitment to understanding these processes across diverse racial and cultural groups, the authors collaborated to center these experiences often underrepresented in mainstream parenting frameworks. She contributed through guidance on conceptualization and study design, feedback during theory development, and contributed to the writing of the manuscript. During theory development and refinement, the authors frequently engaged in discussions of their lived experiences and knowledge about the field to reflect on whether their interpretations held true to the lived experiences of the participants.

### Procedures and participants

This study was approved by the Institutional Review Board at an institution in the Midwestern U.S. Recruitment included flyers in community locations (e.g., restaurants, grocery stores, organizations) and study announcements on email listservs and social media (e.g., Facebook). Interested individuals contacted the research team via email, phone, or text. A team member explained study details and conducted eligibility screening. Eligibility criteria required participants to self‐identify as Hispanic/Latin@/Latinx/Latine, with adolescents aged 11–17 years. To ensure diversity, recruitment included families from various countries of origin and both immigrant and non‐immigrant backgrounds, helping mitigate bias arising from the first author's positionality.

Several interviews were excluded: one dyad (adolescent autism diagnosis), one mother (recording error), and two adolescents who could not be scheduled despite repeated attempts. The final sample included 23 participants: 11 adolescents (*M*_age_ = 14.36 years, SD = 1.77) and 12 mothers (*M*_age_ = 40.08 years, SD = 2.21). Demographic details appear in Table 1 using pseudonyms to protect participants' identities. On average, immigrant adolescents and mothers had lived in the U.S. for 4.63 years (SD = 2.33) and 15.71 years (SD = 8.42), respectively. Mothers' reported origins are reported in Table 1; a majority of mothers were from Mexico (58%). Mothers' education levels included 8.3% elementary, 41.67% middle school, 25% high school, and 25% associate or bachelor's degree.

**TABLE 1:** Demographic information for mothers and teens.

Pseudonym	Age	Race	Country of origin	Nativity	Years in U.S.	Highest level of education
Margarita	41	Not reported	Mexico	Foreign born	16	Middle school
Victor (boy)	12	Not reported	Mexico	Native born	N/A	—
Angela	41	Not reported	Mexico	Foreign born	30	Bachelor's
Carmen (girl)	15	White	Mexico	Native born	N/A	—
Dulce Maria	42	Not reported	Colombia, Mexico	Foreign born	4	Highschool
Beatriz (girl)	16	Not reported	Colombia, Mexico	Foreign born	4	—
Darianny	40	Not reported	Honduras	Foreign born	5	Elementary
Jailene (girl)	17	Not reported	Honduras	Foreign born	5	—
Mercedes	40	Not reported	Mexico	Foreign born	25	Highschool
Estefany (girl)	14	Not reported	Mexico	Native born	N/A	—
Estervina	44	White	Mexico	Foreign born	24	Middle school
Natalie (girl)	14	Not reported	Mexico	Native born	N/A	—
Maitel	35	White	Mexico	Native born	9	Middle school
Virgilio (boy)	14	White	Mexico	Foreign born	8	—
Astrid	42	Not reported	Mexico	Foreign born	3.5	Highschool
Marta (girl)	16	White	Mexico	Foreign born	1.5	—
Yasiris	38	White	Venezuela, Mexico	Foreign born	18	Bachelor's
Naomi (girl)	11	Not reported	Venezuela, Mexico	Native born	N/A	—
Ashley	40	White	Mexico	Foreign born	20	Middle school
Susana (girl)	16	White	Mexico	Native born	N/A	—
Dahianna	40	White	Colombia	Foreign born	14	Middle school
N/A	N/A	N/A	N/A	N/A	N/A	—
Aviarney	38	Not reported	Honduras, Mexico	Foreign born	20	Associate's
Yesenia (girl)	13	White	Mexico	Native born	N/A	—

*Note*: Mother‐adolescent dyads are reported together with mother data reported first, followed by adolescent data. Highest level of education is not reported for adolescents. N/A = the adolescent in this family did not participate.

Interviews were conducted by the first author between November 2023 and November 2024. Interviews were scheduled at participants' convenience and conducted via Zoom in English or Spanish. Consent/assent materials were sent electronically prior to the session, and verbal consent was obtained for recording. Mothers and adolescents were interviewed separately (60–90 min each; ~2 h per family). Each received a $20 electronic gift card and follow‐up materials, including well‐being resources and scholarship information.

### Data collection

#### Semi‐structured interviews

Separate semi‐structured interview protocols (see Supplementary Appendix) were created for Latine mothers and adolescents. The interview protocols were informed by literature on Latine parenting, cultural values, and youth stress and coping, which guided the selection of broad domains, while maintaining open‐ended, neutral, and non‐leading questions. Probes were used to deepen the meaning, detail, and depth of responses. Both protocols provided an opportunity for participants to share their family background, experiences, and cultural values. Additionally, participants were asked to discuss recent interpersonal and academic challenges faced by adolescents, any coping advice mothers may have provided, the coping strategies adolescents used, and thoughts about adolescent mental health.

### Data analysis

All English and Spanish interviews were transcribed, verified, and analyzed in their original language to preserve linguistic and cultural nuance. The first author worked with two bilingual undergraduate research assistants during open coding and initial codebook development. To support their training, she curated key methodological readings (Charmaz, 2014) and facilitated weekly discussions to build conceptual understanding of memoing, open coding, and codebook construction. Following GTM, data collection and analysis occurred concurrently through constant comparison of codes and categories to better understand differences in variations in the data (Charmaz, 2014). The analysis involved three phases: initial coding, intermediate coding, and advanced coding, supported by the qualitative software Taguette (Rampin & Rampin, 2021). Although described sequentially, the process was cyclical and iterative in practice (Charmaz, 2014).

Across three phases of coding, we progressed from descriptive to conceptual and theoretical analysis. In initial coding, the first author and two undergraduate researchers conducted line‐by‐line coding of selected transcripts using gerunds (action‐oriented process codes) and in vivo codes to capture participants' actions and minimize bias, developing a preliminary codebook through constant comparative analysis and memoing (Charmaz, 2014). Importantly, all codes were generated inductively from participants' narratives rather than imposed as a priori categories.

Through intermediate coding, we elevated the analysis by clustering related codes (e.g., listening, being careful, asking questions) into broader categories such as building trust, refining them iteratively through memoing, re‐coding, and concept mapping to clarify their properties and dimensions. This allowed us to be able to relate categories to one another to explain process (Charmaz, 2014). In the advanced coding phase, axial coding, memoing, and diagramming were used to hypothesize relationships between categories. Findings were assessed for theoretical saturation or the point where no new categories or relationships emerge (Charmaz, 2014). Due to time and recruitment limits, full saturation was not achieved, but partial saturation was reached as some properties and dimensions were not represented with sufficient depth or variation to be fully elaborated. But no substantially new categories emerged in later interviews, indicating emerging categories were sufficiently developed to support preliminary theorizing. Finally, a theoretical model was drawn up supported by data excerpts.

### Trustworthiness and credibility

We established trustworthiness of the data through prolonged engagement, iterative coding, and constant comparison to ground findings in data (Charmaz, 2014). Memoing created an audit trail documenting logistics, reflections, and coding decisions while supporting reflexivity on sensitizing concepts. We did not conduct formal member checking, in part to avoid placing additional burden on participants; instead, we relied on these iterative strategies to refine and interrogate our interpretations. Peer debriefing was also employed to ensure the credibility of the findings and involved regular discussions with the two undergraduate research assistants and two colleagues. One colleague is a doctoral candidate with research experience in health disparities and mother education, while the other colleague is a full professor with expertise in grounded theory methods. Both colleagues have a background in Human Development and Family Studies. These collaborative discussions provided external checks and alternative perspectives, enhancing analytic rigor (Creswell & Creswell, 2017).

## RESULTS

The findings from our grounded theory analysis of interviews with Latine mothers and adolescents address how Latine parents socialize youth to cope with interpersonal and academic stressors. The model (see Figure 1) reveals that this process is shaped by cultural values like respeto (respect) and familismo (familism), which frame mothers' understanding of stress and their coping advice. In addition, the analysis highlights the central role of fomentando confianza (building trust) as a foundational relational context, how mothers' awareness of youth stressors prompts them to offer coping advice, and where mothers often draw upon their own prior experiences with similar challenges. Further, the process of aconsejando (advice giving) involves strategies such as prioritizing relational harmony, refocusing expectations, encouraging effort, and redefining agency, aiming to foster youth self‐assurance and equip them with strategic coping skills. Illustrative quotes from mothers demonstrate how cultural and contextual factors influence this socialization process. Because this manuscript centers on mothers' perspectives, adolescents' quotes are used only to provide supporting evidence and context for the emerging categories when applicable. While analysis prioritized responses in participants' preferred language, only English quotes and English translations of Spanish quotes are provided for manuscript brevity. For examples of original quotes in Spanish, see Supplementary Appendix (Table S1). Below, we present each component of the model.

**FIGURE 1:**
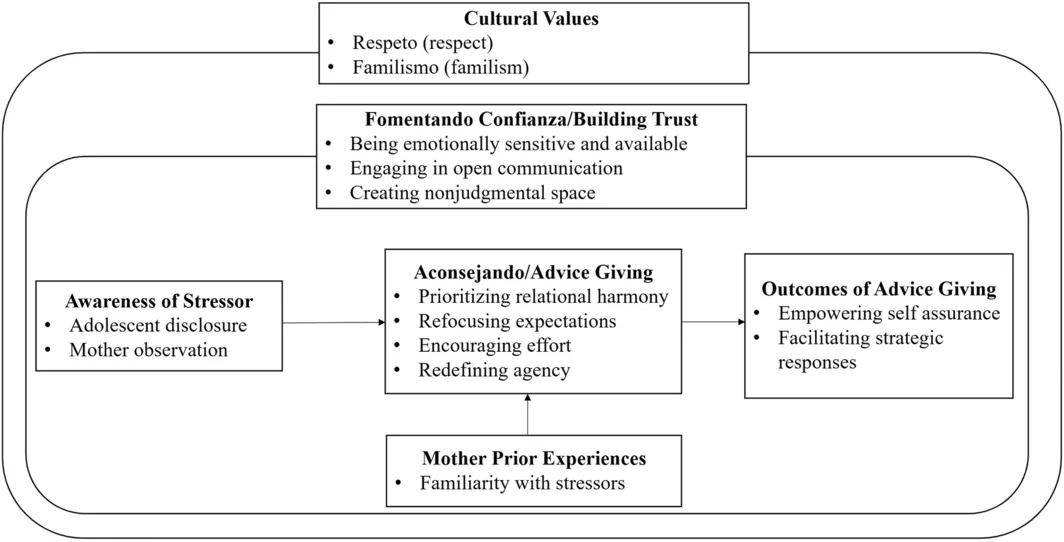
A theoretical model of how Latine mothers guide youth to cope with stress.

## CULTURAL VALUES

Cultural values provide a critical context for understanding how Latine mothers guide youths' development of coping. These values shape mothers' and youths' expectations and behaviors and can affect how families respond to stress. In this study, familismo and respeto emerged as central cultural values through which Latine mothers guide youth to cope with stress.

For familismo, Latine mothers highlight family unity and mutual support as important to well‐being. Mothers view family unity as key to emotional stability, as a responsibility, and as a source of strength that extends beyond the household, even in the face of disagreements. Dulce Maria explained:

Trying to keep the family united … Even though sometimes we think differently, others don't think like we do. That's how we are as a family, trying to understand, not turning our backs. Try to overcome a problem that someone has, and if I can help you, I will help you. But no, I can't turn my back on you.

Respeto was also frequently cited as a core value guiding interpersonal relationships and parenting practices among Latine mothers. Mothers highlight the practical benefits of respeto, noting that it is essential for personal growth and positive social interactions. Darianny shared that disrespecting someone meant that, “They won't like you and they might even turn against you. So you have to have respect, for your family and for other people.” Overall, Latine mothers actively endorse these values and are deeply committed to instilling them in their adolescents.

## FOMENTANDO CONFIANZA (BUILDING TRUST) AS A RELATIONAL CONTEXT

In our study, fomentando confianza functions as a dynamic relational context within which socialization processes occur. Latine mothers build trust by creating an environment in which youth feel secure sharing their concerns and seeking guidance. Building trust requires consistent everyday actions to nurture and sustain a positive mother‐adolescent relationship. These efforts to build trust are expressed through three main strategies outlined below:

### Being emotionally sensitive and available

Latine mothers intentionally use emotional sensitivity and availability as a key strategy for building trust through active listening, asking about their teens' well‐being, showing warmth, and expressing affection. For instance, Margarita explained: “I have shown him affection and respect, not just scolding or telling him off, but TALKING to him.” Mothers were mindful of balancing involvement with respect for autonomy, recognizing that maintaining trust required sensitivity, patience, and dialogue even amid disagreement. As one mother reflected:

Ashley: What I offer her and have given her so far is to talk with her. And at least try to so that she, even if she's not right, I try not to get into a topic that could end up with us fighting. I just tell her that later on she'll understand how things really are. Right now she sees them a certain way, but it's not the right way. Sometimes, when you stumble yourself, you'll understand.

These findings illustrate that Latine mothers' strategies for building trust are rooted in emotional sensitivity, availability, and respeto.

### Engaging in open communication

Mothers used open and consistent communication as a central strategy for building trust via mutual dialogue to maintain closeness, mutual trust, and emotional support. Mothers saw open communication as vital to family functioning, with its absence linked to less respect and more relational strain. Estervina stated:

I think that communication is essential. Especially right now, in this stage, like with Natalie, right?… If there's no communication between us as a couple… or between us and our children, or our children with us, then there's no respect. There's nothing you can do to help them. That's when I feel families start to break down, all their relationships… because you don't have full communication.

However, open communication is not always achieved within families. Some adolescents described relationships characterized by one‐sided or limited dialogue, which hindered the development of trust. This underscores that building trust is a relational process requiring active engagement from both mothers and adolescents. However, in other cases, adolescents acknowledge situations that may limit availability for open communication. Yesenia recognized that work demands and economic hardship limited her mother's availability:

she [my mother] does it every day, going out to work, she endures a lot of humiliation so that I can study here in this country… I think that's a lot, I consider that something she's really done for me. Maybe she hasn't given me enough time, but it's not because she doesn't want to, but because of work and because life here is expensive, costly.

In such cases, trust was sustained through recognition of maternal sacrifice and dedication, reflecting the influence of familismo in shaping how communication and care are understood. Thus, open communication as a strategy is dependent on context and shaped by cultural values and structural constraints. This complexity underscores that building trust is a dynamic negotiated process influenced by both relational practices and contextual realities.

### Creating nonjudgmental space

Latine mothers also build trust by creating a nonjudgmental space for communication, assuring their adolescents that they can share struggles or mistakes without fear of criticism or repercussions. Adolescents felt freer to confide when feedback was delivered with understanding rather than judgment, as one teen explained:

Beatriz: if there are things I've done wrong, she'll tell me, “I think maybe this wasn't right, this wasn't correct,”… but without making me feel judged by her or making me feel like something bad will happen to me later and then I won't tell her.

Mothers reinforce this stance through warmth and reassurance, emphasizing continual support and acceptance. Several mothers framed this nonjudgmental attitude as a parental duty grounded in respect for adolescents' individuality and autonomy. Estervina explained, “The responsibility is ours, to make them feel safe, to ensure they have the full freedom and knowledge, with love and respect, to tell you things… and not be judged.” By prioritizing a nonjudgmental environment, mothers encourage openness and also demonstrate respect for adolescents' individuality in decision‐making.

## AWARENESS OF STRESSOR

Communication about stressors serves as the primary catalyst for mothers to offer coping advice and support. Mothers typically became aware of their adolescents' challenges either through adolescents' direct disclosure or by witnessing the stressor themselves. For example quotes see Supplementary Appendix (Table S1). For adolescent disclosure, many adolescents were forthcoming about their struggles, especially regarding academic challenges because they wanted to do well, while some adolescents viewed it as an obligation.

However, some adolescents felt uncomfortable disclosing due to shame, pride, or fear of upsetting their mothers. Mothers also became aware of stressors through observation or direct experience. Adolescents' reluctance to burden their mothers, paired with maternal efforts to invite communication, illustrates how familismo both constrains and facilitates dialogue. These moments of awareness often marked the beginning of mothers' guidance and coping support.

## MOTHERS' PRIOR EXPERIENCES

Mothers' own life experiences also shaped the coping advice they offered their adolescents. For example quotes see Supplementary Appendix (Table S1). In instances where mothers had little to no direct experiences similar to their adolescents, they tended to emphasize patience and endurance. Limited familiarity with the U.S. education system also constrained some mothers' ability to help. In contrast, mothers with comparable experiences offered more concrete guidance drawn from their own adolescence. Overall, mothers' prior experiences served as a critical filter that shaped how they understood and responded to their adolescents' stressors.

## ACONSEJANDO (ADVICE GIVING)

Latine mothers use consejos (advice) to help adolescents interpret and manage school and interpersonal stressors. Through culturally informed guidance, they aim to strengthen their adolescents' skills. Aconsejando involves providing youth with coping advice through several strategies: (1) prioritizing relational harmony, (2) refocusing expectations, (3) encouraging effort, and (4) redefining agency. Further, rather than relying on a single approach, mothers tailor and combine strategies to fit each situation and the nature of the stressor.

### Prioritizing relational harmony

For interpersonal stress, mothers' guidance reflects values of respeto and familismo, emphasizing reconciliation, forgiveness, and understanding to preserve family bonds. Astrid advised her daughter to reach out to her cousin and make peace: “She's family… try to understand her… forgive her so she doesn't stay stuck in that disagreement, in that anger.” Mothers also encourage empathy by helping youth consider others' perspectives. For example, Jailene recalled her mother reframing conflict: “Sometimes she would say, ‘maybe she [cousin] didn't do it on purpose.’” To protect family harmony, mothers sometimes redirected focus away from hurtful interactions.

These principles extended beyond family relationships. Angela described teaching her adolescents to respond respectfully to peers: “I try to help them so that they can defend themselves, and also so they know they have principles that just as they expect to be respected, they also need to respect others.” By prioritizing relational harmony, mothers reinforced cultural values that promote empathy, peaceful conflict resolution, and responsibility for maintaining positive relationships with family and peers.

### Refocusing expectations

A key strategy Latine mothers use to support coping is refocusing expectations. Rooted in familismo and the high value placed on education, this approach helps adolescents interpret challenges through the lens of family sacrifice and shared goals. Mothers often remind their adolescents that immigration was motivated by the pursuit of better educational opportunities. Astrid explained, “I advise her a lot to make an effort to go to school so she can have a future, that's why we're here, and I advise her to study so she can have a profession.” Education is framed as personal and familial duties. When youth express frustration or consider quitting, mothers reframe difficulties as temporary setbacks. Darianny told her daughter, “You can't drop out [or miss class due to bullying] because this [education] is important for you… these are just little things.”

Mothers also help refocus expectations by normalizing differences in learning and effort. Aviarney explained, “Sometimes some kids don't learn at the same pace… I told her not everyone can be like that.” Such guidance reduces self‐criticism and promotes perseverance.

Refocusing expectations also extended to peer stress. Mothers taught youth not to take unkind remarks personally and to seek supportive friendships. Ultimately, refocusing expectations serves as a protective strategy, helping adolescents develop manageable expectations around peer intentions. Through this strategy, mothers helped adolescents navigate academic setbacks and peer stress with a sense of perspective, self‐worth, and familial support.

### Encouraging effort

Mothers encourage effort by providing coping advice that promotes a proactive approach to both academic and interpersonal challenges. They often advise their adolescents to study, read, and explore topics beyond school requirements to strengthen their independent learning. When youth finished work early or did not feel challenged, mothers encouraged productive use of time. Effort is frequently linked to long‐term goals such as college and career readiness. Mothers normalize obstacles by drawing from their own experiences and framing setbacks as temporary. Beatriz recalled her mother's reassurance that she “went through the same thing… I just needed to put in more effort to get better grades, adapt, and keep improving.”

For interpersonal situations, mothers encourage reaching out to peers, maintaining positive friendships, and adapting when conflicts persist. Aviarney described adjusting her advice after her daughter faced rejection: “I told her to focus on her studies and her friends. At first, I did tell her to get to know those who were bothering her. But when they didn't [respond in a positive way], I told her not to try with them.” By encouraging effort, mothers emphasize perseverance, adaptive problem‐solving, and taking initiative in coping with these stressors.

### Redefining agency

Latine mothers encourage their adolescents to exercise agency in ways that prioritize well‐being, safety, and self‐advocacy. They view avoidance, assertiveness, and help‐seeking as equally empowered forms of agency, each chosen to protect safety and accomplish personal goals. Avoidance is framed as a deliberate self‐protective strategy that helps youth preserve emotional stability and avoid escalation. For instance, Astrid advised her daughter to “ignore it [bullying], don't look for trouble, don't let them expel you… try to adapt.” Such guidance reflects mothers' awareness of the risks of retaliation and their priority on safety over confrontation.

At the same time, mothers encourage self‐advocacy and assertiveness when avoidance no longer suffices. For instance, some mothers promoted assertiveness as a way of protecting their adolescents from further peer victimization. Yasiris told her daughter to “stand firm and say what you need to say… without any physical aggression… right to their face.” Through this framing, assertiveness becomes a means of signaling self‐respect and boundaries without resorting to aggression while also centering respeto and self‐control.

Another example of redefining agency was the encouragement to seek help from trusted adults as a sign of maturity, not weakness. This guidance not only destigmatizes help‐seeking but also aims to empower youth to advocate for themselves within academic settings. Mercedes modeled help‐seeking by recounting her own experience of negotiating with teachers for extra credit or understanding.

I try to tell her, that she has the power to use her words… I tell her, ‘there are teachers who help you in those situations. They'll give you extra work you can do to improve your grade.’ I tell her, ‘I'm telling you from experience. Why? Because I was in high school myself.’

In sum, by redefining agency, mothers provide coping advice that seeks to teach youth to have the wisdom to avoid unnecessary conflict, the courage to assert boundaries and stand up for oneself, and the maturity to seek help when needed. This redefinition of agency aims to provide youth with a flexible set of tools to navigate complex stressors.

## OUTCOMES OF MOTHERS' ADVICE GIVING

Mothers discussed observing or expecting their advice to help adolescents internalize lessons, build confidence (empowering self‐assurance), and manage stress more effectively (facilitating strategic responses). Specifically, as a result of their advice giving, mothers expect youth to develop a stronger sense of self‐assurance so that adolescents can trust their own judgment and abilities when facing challenges. Several described seeing their adolescents grow more confident; for example, Estervina explained, “more relaxed… her conversation is more fluid… more self‐confident, more confident in herself.” Mothers emphasized supporting choices that reflected their adolescents' comfort and readiness. Angela explained, “She should make the best decision [about the advanced class]… she should feel comfortable deciding… I can't do it anymore, I'm going to go back to the previous class.”

Another key outcome that mothers anticipate from their advice giving centers on their adolescents understanding and accepting the guidance, and most importantly, putting it into action. Mothers hope their advice will lead to tangible behavioral changes, such as improved coping with stressors and greater independence in handling difficult situations. Astrid said she wanted her daughter “to understand me… to give me her acceptance… that she's going to try to do it, that I'm right.” Dulce Maria linked change to trust, saying, “That she would change. Since she trusted me, things could really turn out the way I told her they would.” Overall, mothers seek not just compliance but meaningful internalization and application of their guidance.

## DISCUSSION

The findings yielded a grounded theory model that explains how Latine mothers support adolescents in navigating everyday interpersonal and academic stressors. Building upon and extending existing research and theoretical perspectives on Latine parenting in these contexts (Calzada et al., 2010; Hernández & Bámaca‐Colbert, 2016), the model indicates that cultural values such as familismo and respeto shape mother‐adolescent communication, consistent with prior research identifying them as foundational to Latine parenting (Halgunseth, 2019). Latine mothers also prioritize fomentando confianza (building trust) as the basis for providing coping advice. Once aware of their adolescents' stressors, mothers engage in aconsejando (advice giving) through strategies informed by their life experiences. These strategies aimed to scaffold the development of youth self‐assurance and effective coping skills. Connections with cultural values in relation to the components of the model are discussed below.

### Fomentando Confianza (building trust)

The findings illustrate that effective advice giving relies on a trusting mother‐adolescent relationship, which aligns with research highlighting confianza as a cultural value that fosters mutual trust, respect, and strong relational bonds (Bracero, 1998; Halgunseth, 2019). While prior studies have primarily examined communication in Latine families with younger children (Halgunseth, 2019), this work extends those insights to adolescence. Our results echo emic studies showing that Latine mothers view open, positive communication as central to effective parenting during adolescence (Alvarez‐Frank et al., 2025) and with accounts of Cuban American adolescents describing affectionate, involved relationships marked by mutual trust and reciprocity (Crockett et al., 2009). These patterns mirror broader findings in the parenting literature where parents build trust through sensitively attuned parenting practices (Kobak et al., 2017).

In this study, fomentando confianza was essential for both adolescent disclosure and meaningful maternal advice. Without this foundation, youth were less likely to share their stressors, limiting opportunities for support. Reflecting values of interdependence and familismo, some adolescents withheld concerns to avoid burdening parents, a form of forbearance salient in immigrant families seeking to protect parents from added stress (Gonzalez et al., 2020; Martin Romero et al., 2022). These findings underscore that building trust enables effective socialization of coping, whereas adolescent forbearance can constrain these exchanges within Latine families.

### Mothers' strategies for advice giving

Findings reveal that Latine mothers use consejos, culturally rooted advice‐giving practices characterized by wisdom, storytelling, and instructive stories, to support adolescent coping with interpersonal and academic stressors (Delgado‐Gaitan, 1994; Halgunseth, 2019). Mothers tailor advice to both the adolescent's challenges and broader cultural values, drawing on four main strategies: prioritizing relational harmony, refocusing expectations, encouraging effort, and redefining agency. These approaches exemplify adaptive culture, leveraging cultural strengths to meet evolving family needs (García Coll et al., 1996; Hernandez et al., 2024).

Advice promoting relational harmony centered on reconciliation and forgiveness in sibling and cousin conflicts. Consistent with previous studies, this approach reinforces familismo, respeto, perspective taking, and empathy (Bravo et al., 2021; Roos et al., 2024). For academic challenges, mothers in this study helped to refocus their adolescents' expectations by integrating academic socialization and emphasizing education as a family goal. Latine mothers use consejos to set clear academic standards (Aragon, 2018; Holloway et al., 2014), mirroring findings from research in which Latine mothers emphasized education as key to upward mobility (Camacho‐Thompson et al., 2019; Ceballo et al., 2014), especially in transnational families (Hershberg, 2018) and in anti‐immigrant contexts (Philbin & Ayón, 2016).

Encouraging effort centered on persistence and concrete problem‐solving strategies, particularly in academics. Consistent with prior work, mothers urged sustained effort and provided practical support such as time management (Camacho‐Thompson et al., 2019; Delgado‐Gaitan, 1994). This is important given that Latine parent–adolescent discussions of future aspirations have been significantly and positively associated with academic values and school effort (Ceballo et al., 2014). Mothers also extended encouragement to interpersonal contexts, helping youth approach social challenges proactively (McWood et al., 2023).

Redefining agency involved teaching adolescents when to disengage, when to stand up for themselves, and when to seek help. Consistent with prior research, avoidance was framed as a protective strategy against uncontrollable or harmful situations (Martinez et al., 2023). Mothers also promoted non‐violent self‐advocacy, especially when stressors were too intense or persistent to ignore. Similar findings have been found with parents advising youth to confront bullies or advocate for themselves to adults (Cross et al., 2020; Martin Romero et al., 2022). Additionally, mothers encouraged help‐seeking from trusted adults and family (Tulagan et al., 2025). This emphasis on leveraging family resources and resources beyond the family reflects a culturally embedded approach to coping, where collective support is valued and actively mobilized (Kuo, 2013).

Importantly, mothers sometimes encouraged both effort and avoidance (to redefine agency) strategies to address the same situation, advising youth to initially attempt engagement (e.g., resolving a conflict) but to disengage if those efforts were unsuccessful or if the situation became unmanageable (McWood et al., 2023). Taken together, these strategies are developmentally attuned, aligning with literature that highlights adolescence as a period marked by a heightened desire for autonomy and independence (Benito‐Gomez et al., 2020). Latine mothers' strategies reflect an understanding of their adolescents' growing cognitive and socioemotional capacities (e.g., intentionality, metacognition), supporting both the development of autonomy and adaptive coping responses (Zimmer‐Gembeck & Skinner, 2016).

Mothers identified self‐assurance and strategic coping skills as key outcomes of advice giving, aligned with their goals of protecting youth from excessive stress and building a broad coping repertoire (Skinner & Zimmer‐Gembeck, 2016). These findings suggest that cultural values may shape how mothers prioritize these goals, interpret stressors, and determine which coping strategies are most appropriate in different contexts (Darling & Steinberg, 1993).

## LIMITATIONS AND FUTURE RESEARCH

This study's findings should be interpreted in light of several important limitations. First, the sample was composed predominantly of immigrant mothers of Mexican origin, which limits the application of this model to broader Latine populations. Additionally, the focus on immigrant families means the developed categories and relationships may not fully capture experiences of non‐immigrant Latine families. Future grounded theory studies should extend theoretical sampling to refine the properties and dimensions of the categories in the proposed theory. Second, data relied on mother interviews, despite concerted efforts to recruit fathers. This restricts our understanding of the full spectrum of parental SOC and potential for conflicting or complementary advice, as shown in prior studies with distinct parental approaches and gender differences in coping advice (Crockett et al., 2009; McWood et al., 2023).

Additional research on the development and maintenance of trust within Latine families, particularly as it relates to SOC, is also needed. The cultural salience of confianza has received less attention, particularly outside of the education and counseling fields (Bracero, 1998; Teeters et al., 2023). In our data, fomentando confianza functioned as a relational context and was invoked even when mothers did not explicitly discuss confianza as a cultural value. This suggests a need for future work that clarifies or differentiates conceptualizations of how confianza and efforts to build it are understood, as a value, a relational context, or both. For instance, additional theoretical sampling is needed that examines barriers to fomentando confianza and its potential as a protective factor in Latine family contexts. Similarly, future work could also refine conceptual boundaries in the model, particularly for academic contexts where it was more challenging to distinguish advice from expectations.

Theoretical sampling was not feasible due to research constraints, such that new categories or dimensions may still have emerged had additional participants been included. For example, we were not able to explore how mothers prioritize relational harmony in contexts like interactions with teachers or older family members. Future research would be important, as cultural values of respeto and harmony may differentially shape coping advice in hierarchical or intergenerational relationships compared to peer or sibling interactions (Lopez et al., 2023). Although this manuscript centers on mothers' perspectives, analysis of adolescents' interpretations and responses to consejos would further enrich understanding of their role in the SOC process and represents an important direction for future work.

Although our model centers familismo and respeto, future work is needed to examine other cultural values. In our study, other cultural values surfaced, but these categories could not be fully developed. For instance, despite the significance of religion in Latine families (Sanchez et al., 2012), mothers in our study did not discuss religion in their coping advice. Lastly, it will be important to explore unique dynamics within transnational and mixed‐status families, as immigration status influences advice strategies (Cross et al., 2020; Martinez et al., 2023).

## CONCLUSION

The present study advances understanding of how Latine mothers support adolescent coping, utilizing grounded theory methodology to refine existing theories of socialization of coping processes in interpersonal and academic domains. By centering Latine cultural values, this research provides a more culturally grounded perspective on the relational and contextual dynamics that shape Latine mothers' parenting practices. Specifically, the present model identifies fomentando confianza as a relational context that may precede and shape the effectiveness of particular coping messages. The focus on building trust as a foundation for open communication enhances knowledge about how mothers create conditions for youth to disclose stressors. Adolescents may need to experience the parent–adolescent relationship as trusting, safe, and open for advice to be offered and interpreted as supportive. Thus, the relational conditions surrounding coping advice may be as consequential as the content of that advice. Familismo and respeto further shaped this process by informing how mothers encouraged youth to consider relational obligations, manage conflict, and adjust expectations. Cultural values, therefore, may operate not only as content within coping messages but also as relational resources that shape how, when, and under what conditions SOC unfolds. Consistent with calls for culturally responsive family interventions that strengthen communication and relationships (Pineros‐Leano et al., 2023), our model identifies fomentando confianza as a potentially important relational factor that can be used by practitioners to inform intervention components that may support Latine youth well‐being. Additionally, the identification of flexible, culturally informed strategies broadens our understanding of the ways Latine mothers scaffold adolescent coping skills across multiple domains and has implications for considering the motivations underlying mothers' suggestions that could better inform our interpretations about parenting behaviors. However, application of the proposed theory should consider the specific context in which it was developed and whether and how the identified relational processes and parenting strategies may fit similar contexts, rather than assuming broad generalizability. Overall, these findings extend existing frameworks of socialization of coping by highlighting the dynamic, adaptive, and culturally embedded ways that Latine mothers help adolescents manage stress, offering valuable insights that can inform both research and practice.

## AUTHOR CONTRIBUTIONS

**Virnaliz Jimenez:** Conceptualization; investigation; funding acquisition; writing – original draft; methodology; validation; visualization; writing – review and editing; software; formal analysis; project administration; data curation; supervision; resources. **Kelly M. Tu:** Conceptualization; funding acquisition; writing – review and editing; resources; validation; methodology.

## FUNDING INFORMATION

This research was supported through Hatch funding from the U.S. Department of Agriculture's National Institute of Food and Agriculture (ILLU‐793‐307) from the College of Agricultural, Consumer and Environmental Sciences at the University of Illinois Urbana‐Champaign awarded to Kelly M. Tu. This funding supported the study design, data collection, and participant compensation. Any opinions, findings, conclusions, or recommendations expressed in this publication are those of the author(s) and should not be construed to represent any official USDA or U.S. Government determination or policy. This research was also supported by a Dissertation Research Funding award from the Student and Early Career Council of the Society for Research in Child Development awarded to Virnaliz Jimenez, which supported data transcriptions and analysis of data.

## CONFLICT OF INTEREST STATEMENT

The authors have no conflicts of interest to report.

## ETHICS STATEMENT

This study was conducted in accordance with the ethical standards of the APA and was approved by the University of Illinois Urbana‐Champaign Institutional Review Board IRB24‐0905 on 8/22/2023.

## CONSENT STATEMENT

Parent/guardian consent was obtained. Minor assent was obtained.

## Supporting information


**Table S1.** Illustrative quotes of key processes in how latine mothers socialize youth to cope with stressors.

**Data S1.** Supporting Information.

## Data Availability

Research data are not shared.
