# Supplementary material for: Building confianza, sharing Consejos: A grounded theory study of Latine mothers' socialization of coping
Source: J Res Adolesc. 2026 Nov 9;36(4):e70319. doi: 10.1111/jora.70319 (PMC13749057; doi:10.1111/jora.70319)
Supplement: Supplementary file 1 [file JORA-36-0-s001.docx]

Supplementary Appendix

**Supplemental Table 1**

*Illustrative Quotes of Key Processes in How Latine Mothers Socialize Youth to Cope with Stressors*

| Process and Strategies | Spanish Quote | English Translation |
| --- | --- | --- |
| Fomentando Confianza  (Building Trust) | | |
| Being emotionally sensitive and available | Margarita: “He ido demostrarle el cariño, pues el cariño y el respeto, no estarlo como regañando no estarle diciendo, sino que HABARLE.” | “I have shown him affection and respect, not just scolding or telling him off, but TALKING to him.” |
| Engaging in open communication | Estervina: “Yo creo que la comunicación es esencial . . . Este si no la plena comunicación de que te cuente lo realmente no, lo que sucede lo que ya es en su vida diaria. Si no hay comunicación entre nosotros de pareja como yo y mi esposo, nosotros con nuestros hijos, o nuestros hijos con nosotros, entonces no hay ni un respeto . . . O es cuando yo siento que las familias empiezan a quebrar todas sus relaciones o se empieza a desboronar una familia” | “I think that communication is essential . . . If there isn’t full communication where they really tell you what’s happening, what’s actually going on in their daily life if there’s no communication between us as a couple, like between my husband and me, or between us and our children, or our children with us, then there’s no respect . . . That’s when I feel families start to break down, all their relationships, or a family begins to fall apart.” |
| Creating nonjudgmental space | Aviarney: “Más que todo es la confianza. Yo siempre le digo que puedo apoyarla en lo que sea, que me cuente lo que sea, que siempre voy a estar ahí, la voy a apoyar en todo. Que si se siente mal y quiere hablar con alguien pues puede hablar conmigo, que se sienta libre. Que no la voy a juzgar ni nada por el estilo, pues.” | “Above all, it’s about trust. I always tell her that I can support her with anything, that she can tell me anything, that I’ll always be there, I’ll support her in everything. That if she feels bad and wants to talk to someone, she can talk to me, that she should feel free. That I’m not going to judge her or anything like that.” |
|  |  |  |

| Process and Strategies | Spanish Quote | English Translation |
| --- | --- | --- |
| Awareness of Stressor |  |  |
| Adolescent Disclosure | Aviarney: “Decidí ayudarla porque ella desde un principio habló conmigo y me dijo que no entendía [la clase]. A veces era como que mucha información para ella procesarla.” | “I decided to help her because from the beginning she talked to me and told me that she didn’t understand [the class]. Sometimes it was just too much information for her to process.” |
|  | N/A | Victor: “I gave her like a separate like summary of like, the problem [bullying], I didn't give her every single detail. Because it's something that… that I just think she wouldn't quite understand about like how I feel. [I waited to tell her] Uh because I didn't want to worry her about it.” |
| Mother Observation | Dahianna: “[los compañeros] Le dicen por lo menos  ‘llegó el calladito, el que nunca habla.’ Sí, ese tipo de comentarios pues, ‘el tímido’. Este, yo lo escuché, yo misma lo escuché, yo misma, yo misma, con mis propios oídos. ¿Y sabes qué? Normalmente, normalmente cuando uno va para las reuniones, este, bueno uno se entera de algunas cosas pues, pero por boca de mi hijo no.” | “[the classmates] They call him ‘the quiet one, the one who never talks.’ Yes, those kinds of comments, ‘the shy one’. I heard it, I heard it myself, I did, with my own ears. And you know what? Normally, normally when you go to meetings, well, you find out about some things, but not from my son himself.” |
| Mother Prior Experiences | Maitel: “Pues la verdad este a veces me explica de lo que están haciendo en la escuela. Y así dice, ‘ma es que no lo entiendo, no me gusta.’ Y ahora que va a entrar a la high school dice que quiere cambiar de materias. Es que no entiendo la verdad ya muy bien el programa de aquí las escuelas de aquí. Tendría que acercarme más a la escuela. La verdad yo ya no he ido mucho.” | “Well the truth is, sometimes she explains to me what they’re doing at school. And she says, ‘Mom, I don’t understand it, I don’t like it.’ And now that she’s going to start high school, she says she wants to change subjects. Honestly, I don’t really understand the program here the schools here very well. I would need to get closer to the school. Honestly, I haven’t gone much lately.” |
|  |  |  |
| Process and Strategies | Spanish Quote | English Translation |
| Aconsejando  (Advice Giving) |  |  |
| Prioritizing relational harmony | Astrid: “Que su prima no la entiende, que su prima se siente como si fuera una rival, que su prima no la quiere. Yo le aconsejo que es su familia, que debe de hablar con ella, hacer las paces, invitarla a la casa . . . perdonarla para que no se quede ella ahí en ese desacuerdo, en esa rabia que queda ella ahí atascada.” | “That her cousin doesn’t understand her, that her cousin feels like a rival, that her cousin doesn’t like her. I advise her that she’s family, she should talk to her, make peace, invite her to the house . . . forgive her so that she doesn’t stay stuck in that disagreement, in that anger that leaves her stuck there.” |
| Refocusing Expectations | Darianny: “Sí fíjese que eh ella me dijo ‘ya no quiero ir más a la escuela’ me dijo. Ya me ha dicho como tres veces ‘quiero salirme’ me dijo. Entonces le dije yo no te puedes salir porque esto es importante para ti tenés que seguir estudiando. Esto solo son pequeñas cosas y esto te va a servir en un futuro tu estudio.” | “Yes, you know, she told me, ‘I don’t want to go to school anymore.’ She’s told me like three times, ‘I want to drop out.’ So I told her no you can’t drop out because this is important for you, you have to keep studying. These are just little things, and this will help you in the future your education.” |
| Encouraging Effort | Mercedes: “Le digo bueno entonces lo que podrías hacer es no sé practicar tu francés o si lo dejan usar el teléfono vete a tu teléfono si no tienes otra cosa que hacer. O estudia para alguna otra clase que tengas que no se algún examen o algo . . . también podrías hacer algo beneficioso para tus otras clases.” | “I tell her what you could do is, I don’t know, practice your French, or if they let you use your phone, use your phone if you don’t have anything else to do. Or study for another class you have, maybe for a test or something . . . do something beneficial for your other classes ” |
| Redefining Agency | Dulce Maria: “Yo traté de decir que no le hiciera caso. No le hagas caso. Voltea a otro lado. Busca otras amistades. No quiero que te pase nada. Ignórala . . . Trata de evitarla, es la mejor solución. Para no provocar más problemas. ” | “I tried to say that she shouldn’t pay attention to her. Don’t pay attention to her. Look the other way. Find other friends. I don’t want anything to happen to you. Ignore her . . . Try to avoid her, that’s the best solution. So you don’t cause more problems” |
