# Supplementary material for: Building confianza, sharing Consejos: A grounded theory study of Latine mothers' socialization of coping
Source: J Res Adolesc. 2026 Nov 9;36(4):e70319. doi: 10.1111/jora.70319 (PMC13749057; doi:10.1111/jora.70319)
Supplement: Supplementary file 2 [file JORA-36-0-s002.docx]

Supplementary Appendix

Spanish Parent Interview Protocol

**Protocolo de Entrevista a Padres**

Introducción: Hola. Mi nombre es ______ y soy estudiante de posgrado en la Universidad. Gracias por tomarse el tiempo de hablar conmigo hoy. Estoy haciendo entrevistas para explorar cómo los padres y jóvenes latinos hablan sobre el estrés interpersonal y académico. Le preguntaré sobre su historia, experiencias personales y conversaciones que tenga con su hijo sobre la escuela y sus relaciones. Espero aprender de usted acerca de sus experiencias y pensamientos---no hay respuestas correctas o incorrectas.

Esta entrevista durará entre una hora y una hora y media. Su participación es voluntaria y puede omitir las preguntas que no desea responder. Su información permanecerá privada. Nunca usaré tu nombre real, ¿qué nombre te gustaría elegir para ti?

Me gustaría grabar la entrevista para asegurarme de que entiendo correctamente lo que has compartido. No compartiré la grabación con su hijo. ¿Te parece bien que grabe la entrevista? ¿Tienes alguna duda antes de empezar?

**Antecedentes familiares**

Para ayudarnos a iniciar nuestra conversación...

1. ¿A quién consideras tu familia? ¿Dónde vive cada uno de estos miembros de la familia?
2. ¿Puedes hablarme de los orígenes/antecedentes *[país de origen]* de su familia?
3. ¿Dónde creciste?
   a. ¿Cómo fue crecer allí?
4. ¿Qué tan conectado te sientes con [*país de origen*]?
   a. Pide ejemplos

**Socialización de padres sobre el manejo del estrés**

Gracias por compartir información sobre su familia y experiencias conmigo. Ahora me gustaría que piense en [*adolescente*].

5. ¿Cómo describirías tu relación con [*adolescente*]?
6. En un día común, ¿de qué sueles hablar tú y [*adolescente*]?

Es común que los adolescentes enfrenten situaciones estresantes durante esta etapa de su vida.

7. ¿Cuáles son los tipos de estrés que experimenta [*adolescente*]?
8. Normalmente, ¿cómo le ayudas cuando está pasando por esas situaciones estresantes?

**Factores estresantes interpersonales**

Ahora, quiero que pienses en experiencias que *[adolescente]* haya tenido con problemas en sus relaciones. Por ejemplo, un momento en el que *[adolescente]* tuvo problemas con otra persona, tal vez familiares, amigos, compañeros de clase, maestros u otras personas.

9. ¿Qué tipo de desafíos interpersonales ha experimentado [*adolescente*] en el último año?
10. Hay alguna otra experiencia/ejemplo que te venga a la mente?
11. ¿Cómo ha afectado [*factor estresante*] a [*adolescente*]?
    a. ¿Cómo afectó [*factor estresante*] el nivel de estrés de [*adolescente*]?
    b. ¿Cómo afectó [*factor estresante*] el estado de ánimo de [*adolescente*]?
    c. Probe – la alimentación, el sueño y otros comportamientos
12. ¿Puedes hablarme sobre un ejemplo reciente de cuando estabas hablando sobre un desafío interpersonal con [*adolescente*]? (es decir, esto podría ser un problema con un compañero, familiar u otro adulto)
    a. ¿Cuéntame de qué hablaste con [*adolescente*]?
       i. ¿Cuánto conoces a [*persona/personas involucradas en el problema*]?
    b. Cuéntame más sobre cualquier cosa que hayas hecho o dicho para ayudarle a enfrentar la situación.
       i. Explora más-- ¿Hay algo más que hayas hecho o dicho para ayudar a [*adolescente*] con este desafío?
    c. ¿Cómo decidiste [*respuesta del padre*] en respuesta al desafío de [*adolescente*]?
       i. [*Pregunta por cada respuesta que mencionaron*]
    d. ¿Qué esperabas que sucediera cuando [*respuesta del padre]*?
       i. [*Pregunta por cada respuesta que mencionaron*]
13. ¿Cómo respondió [*adolescente*] cuando tu [*respuesta del padre*]?
    a. Explora más--¿sentimientos de tristeza o nervios? ¿Sentimientos de ira? ¿Otros sentimientos?
    b. [*Pregunta por cada respuesta que mencionaron*]
14. ¿Qué señales buscas para entender el bienestar de [*adolescente*] cuando se trata de sus relaciones?
15. ¿Con qué frecuencia suelen tener conversaciones en tu familia sobre el bienestar al lidiar con desafíos interpersonales?

**Estresores académicos**

Genial, ahora me gustaría saber sobre las experiencias escolares de *[adolescente].*

16. Cuéntame sobre la escuela de [*adolescente*].
    a. Explora más--¿ Cómo es allí? ¿Cómo es la escuela?
    b. Explora más--¿ ¿Qué relación tienes con la escuela? ¿Maestros? ¿Director? ¿Administradores?

Ahora, quiero que pienses en experiencias que [*adolescente*] haya tenido con desafíos académico. Por ejemplo, podría ser un momento en el que *[adolescente]* tuvo problemas para completar la tarea, no entendió algo, estuvo aburrido con la escuela, se sintió presionado para sacar buenas notas en los exámenes.

17. **¿**Qué dificultades académicas ha experimentado [*adolescente*] durante el año escolar más reciente?
    a. ¿Hay alguna otra experiencia/ejemplo que te venga a la mente?
18. ¿Cómo ha afectado [*factor estresante*] a [*adolescente*]?
    a. ¿Cómo afectó [*factor estresante*] el nivel de estrés de [*adolescente*]?
    b. ¿Cómo afectó [*factor estresante*] el estado de ánimo de [*adolescente*]?
    c. Explora más --- la alimentación, el sueño y otros comportamientos
19. ¿Puedes hablarme sobre un ejemplo reciente de cuando estabas hablando sobre un desafío académico con *[adolescente*]? (es decir, esto podría ser un problema con tareas, exámenes, aburrirse con la escuela, no entender algo o mantenerse al día con las tareas escolares)
    a. ¿Cuéntame de qué hablaste con [*adolescente*]?
       i. Explora más---¿Cuánto conoces a [*persona/personas involucradas en el problema*]?
    b. Cuéntame más sobre cualquier cosa que hayas hecho o dicho para ayudarle a enfrentar la situación.
       i. Explora más-- ¿Hay algo más que hayas hecho o dicho para ayudar a [*adolescente*] con este desafío?
    c. ¿Cómo decidiste [*respuesta del padre*] en respuesta al desafío de [*adolescente*]?
       i. [*Pregunta por cada respuesta que mencionaron*]
    d. ¿Qué esperabas que sucediera cuando [*respuesta del padre]*?
       i. [*Pregunta por cada respuesta que mencionaron*]
20. ¿Cómo respondió [*adolescente*] cuando tu [*respuesta del padre*]?
    a. Explora más--¿sentimientos de tristeza o nervios? ¿Sentimientos de ira? ¿Otros sentimientos?
    b. [*Pregunta por cada respuesta que mencionaron*]
21. ¿Qué señales busca para entender el bienestar de [*adolescente*] cuando se trata de la escuela?
22. ¿Con qué frecuencia suelen tener conversaciones en tu familia sobre el bienestar al lidiar con desafíos académicos?

**Valores culturales latinos**

Gracias por compartir sus experiencias y conversaciones que ha tenido con su hij@. En esta última parte, quiero cambiar completamente de tema para conocer más sobre su cultura.

23. Pensando en tus propios orígenes/antecedentes y experiencias, ¿qué significa para ti ser *[país de origen]*?
    a. ¿Qué valores (por ejemplo, ideas, creencias) son importantes para usted como padre *[país de origen]*?

*Diga: "Los valores pueden ser cosas como creencias sobre la familia, la religión, la escuela, el trabajo, los roles de género, las relaciones, el dinero, cómo las personas se relacionan entre sí, las reglas sociales, cosas así".*

b. ¿Por qué crees que son importantes para ti?
c. [*Pregunte por cada valor/idea proporcionada*] ¿Puedes hablarme de un momento en el que ese valor se sintió muy fuerte para usted?
d. [*Pregunte por cada valor/idea proporcionada*] ¿Puedes hablarme de un momento en el que este valor guió la crianza de tus hijos? (*…tu manera de ser padre?)*

24. ¿Qué valores crees que es importante transmitir a tu hij@?
25. ¿Cómo crees que los valores culturales influyen, si es que lo hacen, la manera en que ayudas a [*adolescente*] con cosas estresantes?

Gracias por compartir sus valores, las experiencias de su hij@ y las formas en que le ha hablado sobre los desafíos.

26. Si pudieras compartir algún consejo con otros padres, ¿qué consejo le darías a los padres latinos que quieren ayudar a sus hijos a lidiar con estrés interpersonales y académicos?

**Reflexiones finales**

Esas son todas las preguntas que tengo para ti. ¿Hay algo más sobre tu experiencia que no hayamos cubierto en esta entrevista que creas que es importante que yo (u otros) sepamos? ¿Tienes alguna pregunta para mí?

Antes de terminar, quería mencionar dos cosas más. Cuando hablamos de nuestros hallazgos, queremos ser respetuosos con las diferentes identidades. Entonces, tengo un par de preguntas demográficas que hacerles. Estos son opcionales.

27. ¿Cuál es tu género? (¿Qué pronombres prefieres?)
28. ¿Con qué raza(s) te identificas?
    a. **Asiático**
       i. Chino, vietnamita, filipino, coreano, japonés, indio asiático, paquistaní, camboyano, hmong, etc.
       ii. Otro:
    b. **Indio americano o nativo de Alaska**
       i. Indios americanos, nativos de Alaska, indios de América Central o del Sur
       ii. Nación Navajo, Tribu de los Pies Negros, Mayas, Aztecas, etc.,
       iii. Otro:
    c. **Negro**
       i. Afroamericano, jamaiquino. nigerianos, etíopes, haitianos, somalíes, ghaneses, sudafricanos, barbadenses, etc.,
       ii. Otro:
    d. **Oriente Medio o Norte de África**
       i. libaneses, sirios, iraníes, marroquíes, egipcios, argelinos, israelíes, iraquíes, kurdos, etc.,
       ii. Otro:
    e. **Blanco**
       i. alemán, italiano, inglés, irlandés, polaco, francés, escocés, noruego, holandés, etc.,
       ii. Otro:
    f. **Nativo de Hawái o de las islas del Pacífico**
       i. nativos de Hawái, samoa, tonga, fiji, chamorro, marshallese, palauano, tahitiano, chuukese, etc.,
       ii. Otro:
29. ¿Cuál es el nivel más alto de educación que has completado?
30. ¿A qué te dedicas actualmente? / ¿En qué trabajas actualmente?

Con el fin de garantizar que recibas el pago,

- $20 a través de una tarjeta de regalo: ¿cuál es la mejor dirección de correo electrónico para enviarle la tarjeta de regalo de $20?

Ok ¡gracias por su participación!

Muchas gracias por tomar el tiempo de responder a mis preguntas. Dejaré de grabar ahora.

| **Preguntas comunes o palabras de aliento**  Nota: Escuche las palabras o frases repetidas que parezcan tener un significado y pida al participante que aclare el significado. | | |
| --- | --- | --- |
| ¿Puedes contarme más sobre eso? | ¿Puedes darme un ejemplo? | ¿Cómo fue eso? |
| Eso es interesante. | Eso tiene sentido. | Me estás dando información útil. |

English Parent Interview Protocol

**Parent Interview Protocol**

Introduction: Hello. My name is ______, and I am a graduate student at the University. Thank you for taking the time to talk with me today. I am doing interviews to explore how Latino parents and youth talk about interpersonal and academic stress. I will be asking you about your background, personal experiences, and conversations you have with your child about school and their relationships. I hope to learn from you about your experiences and thoughts—there are no right or wrong answers.

This interview will take about an hour to an hour and a half. Your participation is voluntary, and you can skip questions you don’t want to answer. Your information will remain private. I will never use your real name—what name would you like to choose for yourself?

I would like to record the interview so that I can make sure that I get down what you’ve shared correctly. I will not share the recording with your child. Is it okay with you if I record the interview? Do you have any questions before we start?

**Background Information**

To help us start our conversation…

1. Who do you consider to be your family? Where do each of these family members live?
2. Can you tell me about your family’s *[country of origin]* background?
3. Where did you grow up?
   a. What was it like growing up there?
4. How connected do you feel with [*country of origin*]?
   a. Probe: ask for examples

**Socialization of Coping**

Thank you for sharing information about your family and background with me. Now I’d like for you to think about [*teen*].

5. How would you describe your relationship with *[teen]*?
6. On a typical day what do you and *[teen]* usually talk about?

It’s common for teens to deal with stressors during this time in their life.

7. What are the kinds of stress that [*teen*] experiences?
8. How do you usually help them when they are going through those stressors?

**Interpersonal Stressors**

Now I would like you to think about any experiences *[teen]* has had with challenges in their relationships. For example, this could be a time when they had troubles with family, friends, classmates, teachers, or others.

9. What kind of interpersonal challenges has *[teen]* experienced within the last year?
   a. Are there any other experiences/examples that come to mind?
10. How has *[specific stressor]* affected [*teen*]?
    a. How did *[specific stressor]* impact [*teen*]’s level of stress?
    b. How did *[specific stressor]* impact [*teen*]’s mood?
    c. Probe for– eating, sleep, other behaviors
11. Can you tell me about a recent example of when you were discussing an interpersonal challenge with *[teen]*? (i.e., this could be an issue with *peer, family, or other adult*)
    a. Tell me about what you talked about.
       i. Probe: How well do you know [*person/people involved in problem]?*
    b. Tell me more about anything you did or said to help them deal with the situation.
       i. Probe: Is there anything else you did or said to help [*teen*] with this challenge?
    c. How did you decide to [*parents response*] in response to [*teen’s*] challenge?
       i. [*Ask for each response they mentioned*]
    d. What were you hoping would happen when you [*parents response*]?
       i. [*Ask for each response they mentioned*]
12. How did *[teen]* respond to your [*parents response*]?
    a. Probe: feelings of sadness or feeling nervous? feelings of anger? Other feelings?
    b. [*Ask for each response they mentioned*]
13. What signs do you look for to understand [*teen*]’s well-being when it comes to relationships?
14. How often does your family have conversations about well-being when dealing with interpersonal challenges?

**Academic Stressors**

Great, now I would like to learn about [*teen’s*] school experiences.

15. Tell me about [*teen’s*] school.
    a. Probe: What is it like there?
    b. Probe: What is your relationship to the school? Teachers? Principal? Administrators?

Next, I want to discuss any troubles *[teen]* has had with academic challenges. For example, this could be a time when they had trouble completing homework, not understanding something, being bored with school, feeling pressure to do well, not doing well on tests.

16. What kind of academic challenges has *[teen]* experienced within the most recent school year?
    a. Are there any other experiences/examples that come to mind?
17. How has *[specific stressor]* affected [*teen*]?
    a. How did *[specific stressor]* impact *[teen]*’s level of stress?
    b. How did *[specific stressor]* impact *[teens]*’s mood?
    c. Probe for– eating, sleep, other behaviors
18. Can you tell me about a recent example of when you were discussing an academic challenge with *[teen*]? (i.e., this could be an issue with *homework, tests, being bored with school, not understanding something, or keeping up with schoolwork*)
    a. Tell me about what you talked about.
       i. Probe only if relevant: How well do you know [*person/people involved in problem*]?
    b. Tell me more about anything you did or said to help them deal with the situation.
       i. Probe: Is there anything else you did or said to help [*teen*] with this challenge?
    c. How did you decide to [*parents response*] in response to [*teen’s*] challenge?
       i. [*Ask for each response they mentioned*]
    d. What were you hoping would happen in [*parents response*]?
       i. [*Ask for each response they mentioned*]
19. How did *[teen]* respond to your advice about *[specific stressor]*?
    a. Probe: feelings of sadness or feeling nervous? feelings of anger? Other feelings?
    b. [*Ask for each response they mentioned*]
20. What signs do you look for to understand *[teen]*’s well-being when it comes to school?
21. How often does your family have conversations about well-being when dealing with academic challenges?

**Cultural Values**

Thank you for sharing your experiences and conversation with your child. In this last section, I want to change topics completely to learn more about your cultural background.

22. Thinking about your own background and experiences, what does it mean to you to be [*country of origin*]?

a. What values (e.g., ideas, beliefs) are important to you as a [*country of origin*] parent?

*Say – “Values can be things like beliefs about family, religion, school, work, gender roles, relationships, money, how you relate to one another, social rules, things like that.”*

a. [*Ask for each value/idea provided]* Could you tell me about a time when that value felt very strong for you?
b. [*Ask for each value/idea provided*] Could you tell me about a time when this value guided your parenting?

23. What values do you think are important to pass on to your teen?

Thank you for sharing your values, your teens’ experiences, and the ways you’ve talked to them about challenges.

24. If you could share some advice with other parents, what advice do you have for Latino parents who want to help their children deal with interpersonal and academic challenges?

**Final Thoughts**

Those are all the questions that I have for you. Is there anything else about your experience that we have not covered in this interview that you feel is important for me (or others) to know? Do you have any questions for me?

Before we finish, I wanted to mention two more things. When we talk about our findings, we want to be respectful of different identities. So, I have a couple demographic questions to ask you. These are optional.

1. What is your gender? (What pronouns do you prefer?)
2. What race(s) do you identify with?
   a. **Asian**
      i. Chinese, Vietnamese, Filipino, Korean, Japanese, Asian Indian, Pakistani, Cambodian, Hmong etc.,
      ii. Other:
   b. **American Indian or Alaska Native**
      i. American Indian, Alaska Native, Central or South American Indian
      ii. Navajo Nation, Blackfeet Tribe, Mayan, Aztec, etc.,
      iii. Other:
   c. **Black**
      i. African-American, Jamaican. Nigerian, Ethiopian, Haitian, Somali, Ghanaian, South African, Barbadian etc.,
      ii. Other:
   d. **Middle Eastern or North African**
      i. Lebanese, Syrian, Iranian, Moroccan, Egyptian, Algerian, Israeli, Iraqi, Kurdish etc.,
      ii. Other:
   e. **White**
      i. German, Italian, English, Irish, Polish, French, Scottish, Norwegian, Dutch etc.,
      ii. Other:
   f. **Native Hawaiian or Pacific Islander**
      i. Native Hawaiian, Samoan, Tongan, Fijian, Chamorro, Marshallese, Palauan, Tahitian, Chuukese etc.,
      ii. Other:
3. What is your highest level of education completed?
4. What is your current occupation?

In order to ensure you receive payment,

- $20 via Gift card – what is the best email address to use to send you the $20 gift card?

Again, thank you for your participation!

Thank you so much for taking the time to answer my questions. I will stop recording now.

| **Common Probes or Words of Encouragement**  Note: Listen for repeated words or phrases that seem to carry meaning and ask participant to clarify meaning. | | |
| --- | --- | --- |
| Can you tell me more about that? | Can you give me an example? | What was that like? |
| That is interesting. | That makes sense. | You’re giving me helpful information. |
